# Synchronous occurrence of oral squamous cell carcinoma and aggressive papillary thyroid microcarcinoma with synchronous regional metastasis in the cervical lymph nodes: a case report

**DOI:** 10.1093/jscr/rjag616

**Published:** 2026-07-21

**Authors:** Dávid Ágoston Kovács, Miklós Bodor, Insha Mukhtar, Sándor Barna, László Tóth, Róbert Boda, Sándor Kovács, Dezső Tóth, Ferenc Győry

**Affiliations:** University of Debrecen, Faculty of Medicine, Department of Surgery, Móricz Zsigmond krt. 22, H-4032 Debrecen, Hungary; University of Debrecen, Faculty of Medicine, Department of Internal Medicine, Nagyerdei krt. 98, H-4032 Debrecen, Hungary; Ashford and St Peters' NHS Foundation Trust, Department of Geriatric Medicine, Guildford Road, Chertsey KT16 0PZ, United Kingdom; University of Debrecen, Scanomed Ltd, Nagyerdei krt. 98, H-4032 Debrecen, Hungary; University of Debrecen, Faculty of Medicine, Department of Pathology, Nagyerdei krt. 98, H-4032 Debrecen, Hungary; University of Debrecen, Faculty of Medicine, Department of Maxillofacial Surgery, Nagyerdei krt. 98, H-4032 Debrecen, Hungary; University of Debrecen, Faculty of Business and Economics, Coordination and Research Centre for Social Sciences, Böszörményi út 138, H-4032 Debrecen, Hungary; University of Debrecen, Faculty of Medicine, Department of Surgery, Móricz Zsigmond krt. 22, H-4032 Debrecen, Hungary; University of Debrecen, Faculty of Medicine, Department of Surgery, Móricz Zsigmond krt. 22, H-4032 Debrecen, Hungary

**Keywords:** papillary thyroid microcarcinoma, papillary thyroid carcinoma, thyroid carcinoma, floor of the mouth carcinoma, synchronous regional metastasis, clinical practice change, diagnostic vigilance

## Abstract

This report describes an extremely rare case of synchronous oral squamous cell carcinoma (OSCC) and aggressive papillary thyroid microcarcinoma (PTMC) presenting with synchronous regional metastasis to the cervical lymph nodes. A 56-year-old male underwent excision of a floor-of-mouth squamous cell carcinoma and supra-omohyoid neck dissection. Histopathology unexpectedly revealed distinct metastatic components of both OSCC and PTMC within the same lymphatic basin, despite unremarkable preoperative thyroid imaging. Total thyroidectomy subsequently confirmed bilateral microscopic PTMC (0.1 mm) with skip metastases to the lateral neck. To avoid radiation-induced fibrosis that would complicate thyroid surgery, thyroidectomy was prioritized over adjuvant radiotherapy for the oral malignancy. This case challenges the perception of PTMC as an indolent disease and highlights the need for multidisciplinary surgical sequencing and high diagnostic vigilance when managing dual head and neck malignancies.

## Introduction

Thyroid carcinoma is the most common endocrine malignancy, with papillary thyroid carcinoma (PTC) being the predominant subtype [1]. Papillary thyroid microcarcinoma (PTMC), defined by a diameter ≤ 10 mm, is often managed conservatively due to its typically indolent nature [1, 2]. However, thyroid malignancies are incidentally discovered in 0.3% to 1.9% of patients undergoing surgery for head and neck squamous cell carcinoma (HNSCC) [3]. The synchronous occurrence of oral squamous cell carcinoma (OSCC) and PTMC with regional metastasis is an academic and clinical rarity [4]. Such cases require high radiological vigilance, as these synchronous primary tumors are often detected incidentally during staging [5]. While the pathogenesis remains debated—ranging from coincidence to shared environmental risk factors—these subclinical malignancies present complex diagnostic conundrums when they exhibit aggressive metastatic behavior [6]. Literature regarding the co-occurrence of OSCC and PTMC within the same lymphatic basin distinguishes between two distinct entities: (i) Collision Metastasis, where histologically distinct malignant cells from both primaries are intertwined within a single lymph node [7, 8], and (ii) Synchronous Regional Metastasis, where distinct nodes harbor different primary origins, as seen here.

## Case report

A 56-year-old male with a history of hypertension presented with a moderately differentiated squamous cell carcinoma of the floor of the mouth (pT1). Preoperative neck ultrasound was negative for thyroid or nodal pathology. The patient underwent intraoral tumor excision and supra-omohyoid neck dissection (SOHD). Post-operative histology of the SOHD specimens revealed a complex metastatic profile:

- Level I-III: One lymph node contained metastatic squamous cell carcinoma (p40+), while a separate, distinct lymph node contained metastatic papillary thyroid carcinoma (CK19+).
- Parajugular nodes: A 0.5 cm metastatic PTMC deposit was identified.
- No ‘collision metastasis’ was found; rather, these were synchronous regional metastases.

**Figure 1:**
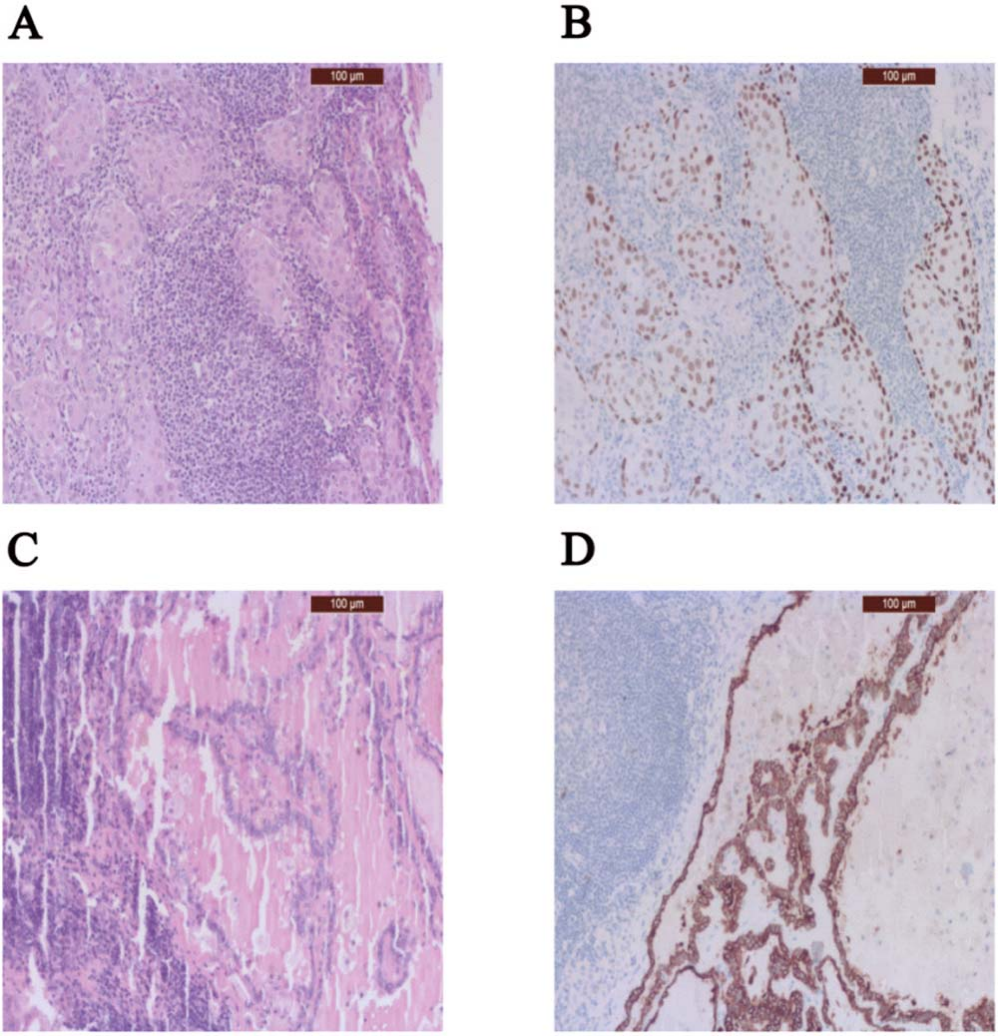
Synchronous regional metastasis of OSCC and PTMC. (A) H&E staining of metastatic OSCC showing squamous cell nests with keratinization. (B) Immunohistochemical p40+ expression confirming the squamous lineage. (C) H&E staining of metastatic PTMC exhibiting characteristic papillary architecture. (D) Immunohistochemical staining (CK19+) demonstrating the distinct epithelial profile of the thyroidal component. *Note: The images highlight the morphological and immunohistochemical divergence of these synchronous metastatic populations within the cervical lymphatic basin. Original magnification ×200.*

The diagnostic findings were confirmed by immunohistochemical profiling, as detailed in Fig. 1. The OSCC component expressed p40 and was Thyroglobulin negative, while the PTMC component demonstrated intensive CK19 positivity, alongside focal Galectin-3 and HBME-1 expression.

The patient subsequently underwent total thyroidectomy and bilateral modified radical neck dissection (MRND). Histology confirmed bilateral multifocal PTMC with the largest foci measuring an extraordinarily small 0.1 mm (pT1a(m)). Despite the microscopic size of the primary thyroid tumors, they had produced regional metastases up to 0.5 cm. Central compartment nodes (0/16) were negative, confirming a ‘skip metastasis’ pattern to the lateral neck [9].

To avoid radiation-induced fibrosis complicating the thyroid surgery, the team prioritized the total thyroidectomy over adjuvant radiotherapy for the OSCC. Following surgery and radioiodine therapy (3700 MBq of 131I), the patient remained disease-free. He has reached a 5-year follow-up period without recurrence of either malignancy.

## Discussion

This case highlights the potentially aggressive behavior of PTMC, challenging the paradigm that sub-millimeter lesions are universally indolent [1, 2]. It represents a critical advance in understanding dual head and neck malignancies, where synchronous tumors obscure diagnosis [3, 4]. A high index of suspicion is essential during oncologic workup [3, 5]. As shown in the histological evidence in Fig. 1, the dissociation between primary tumor size (0.1 mm) and metastatic potential (0.5 cm) is striking. Standard risk factors for metastasis include male gender and size >5 mm, but this case suggests that tumor biology may outweigh size [10, 11]. The presence of lateral cervical involvement without central node disease confirms the ‘skip metastasis’ pattern, which occurs in approximately 19.7% to 22% of PTC cases [9]. Such patterns explain why preoperative imaging often fails to detect occult micro-metastases [12, 13]. Pathological differentiation was achieved via immunohistochemistry; CK19, Galectin-3, and HBME-1 confirmed thyroid origin, while p40 confirmed squamous differentiation [11]. Regarding management, prioritizing thyroid surgery before radiotherapy minimized surgical morbidity and ensured oncological radicality in a complex clinical scenario [14].

## Conclusion

The synchronous occurrence of OSCC and PTMC with regional metastasis is a rare entity that fundamentally challenges the traditional view of PTMC as an invariably indolent disease. This case demonstrates that even microscopic thyroid carcinomas as small as 0.1 mm can exhibit aggressive behavior. In complex synchronous presentations, prioritizing tailored surgical sequencing—specifically thyroid surgery prior to adjuvant radiotherapy—is a safe and effective strategy to ensure complete disease clearance.
